# Designing malaria surveillance strategies for mobile and migrant populations in Nepal: a mixed-methods study

**DOI:** 10.1186/s12936-019-2791-1

**Published:** 2019-05-03

**Authors:** Jennifer L. Smith, Prakash Ghimire, Komal Raj Rijal, Alysse Maglior, Sara Hollis, Ricardo Andrade-Pacheco, Garib Das Thakur, Nabaraj Adhikari, Upendra Thapa Shrestha, Megha Raj Banjara, Bibek Kumar Lal, Jerry O. Jacobson, Adam Bennett

**Affiliations:** 1 Malaria Elimination Initiative, Global Health Group, University of California, San Francisco, USA; 2 Central Department of Microbiology, Tribhuvan University, Kirtipur, Kathmandu Nepal; 3 Epidemiology and Diseases Control Division, Ministry of Health and Population, Teku, Kathmandu Nepal

**Keywords:** Imported malaria cases, Mobile migrant populations (MMPs), Focus group discussion, Key informants’ interview, Malaria elimination, Nepal

## Abstract

**Background:**

As malaria cases have declined throughout Nepal, imported cases comprise an increasing share of the remaining malaria caseload, yet how to effectively target mobile and migrant populations (MMPs) at greatest risk is not well understood. This formative research aimed to confirm the link between imported and indigenous cases, characterize high-risk MMPs, and identify opportunities to adapt surveillance and intervention strategies to them.

**Methods:**

The study used a mixed-methods approach in three districts in far and mid-western Nepal, including (i) a retrospective analysis of passive surveillance data, (ii) a quantitative health facility-based survey of imported cases and their MMP social contacts recruited by peer-referral, and (iii) focus group (FG) discussions and key informant interviews (KIIs) with a subset of survey participants. Retrospective case data were summarised and the association between monthly indigenous case counts and importation rates in the previous month was investigated using Bayesian spatio-temporal regression models. Quantitative data from structured interviews were summarised to develop profiles of imported cases and MMP contacts, including travel characteristics and malaria knowledge, attitudes and practice. Descriptive statistics of the size of cases’ MMP social networks are presented as a measure of potential programme reach. To explore opportunities and barriers for targeted malaria surveillance, data from FGs and KIIs were formally analysed using a thematic content analysis approach.

**Results:**

More than half (54.1%) of malaria cases between 2013 and 2016 were classified as imported and there was a positive association between monthly indigenous cases (incidence rate ratio (IRR) 1.02 95% CI 1.01–1.03) and the previous month’s case importation rate. High-risk MMPs were identified as predominantly adult male labourers, who travel to malaria endemic areas of India, often lack a basic understanding of malaria transmission and prevention, rarely use ITNs while travelling and tend not to seek treatment when ill or prefer informal private providers. Important obstacles were identified to accessing Nepali MMPs at border crossings and at workplaces within India. However, strong social connectivity during travel and while in India, as well as return to Nepal for large seasonal festivals, provide opportunities for peer-referral-based and venue-based surveillance and intervention approaches, respectively.

**Conclusions:**

Population mobility and imported malaria cases from India may help to drive local transmission in border areas of far and mid-western Nepal. Enhanced surveillance targeting high-risk MMP subgroups would improve early malaria diagnosis and treatment, as well as provide a platform for education and intervention campaigns. A combination of community-based approaches is likely necessary to achieve malaria elimination in Nepal.

**Electronic supplementary material:**

The online version of this article (10.1186/s12936-019-2791-1) contains supplementary material, which is available to authorized users.

## Background

As a result of sustained control measures, the malaria burden in Nepal has declined steadily since 1985 and malaria transmission has reached a very low level, with an annual parasite incidence (API) of 0.04 cases per 1000 population in 2016 [1]. Although Nepal is moving towards implementing elimination strategies, high rates of importation along its southern border present a major challenge to continued progress. As the number of total cases declined by 92% between 2002 (12,750 cases) and 2016 (1009 cases), the proportion of cases classified as imported increased from approximately 20 to 50% [2]. The majority of imported cases during this period reported a history of travel to malaria-endemic areas of India. Close to two million Nepalese are estimated to work in India as seasonal or long-term labourers, and a large proportion of external labour migration, particularly seasonal migration, is believed to originate in malaria endemic areas such as far- and mid-western Nepal [3–5]. This population movement across Nepal’s southern border, which citizens of both nations may cross freely, is thought to contribute to persistence of local malaria foci in border areas [5–7]. The fact that imported cases have not declined at the same rate as indigenous cases suggests that additional control measures may be needed to effectively prevent, detect and resolve infections among mobile and migrant populations (MMPs).

Free malaria testing and treatment are available to Nepali and Indian nationals through 162 government primary health care (PHC) centres, health posts (HPs) and sub-health posts (SHPs) in 22 border districts of Nepal [8]. The standard of care includes radical cure for *Plasmodium vivax* consisting of a 14-day course of primaquine in combination with chloroquine [9]. During the eradication era (1960–1970), the National Malaria Control Programme (NMCP) of Nepal operated cross-border screening in posts in Kakarvitta of Jhapa District in east and Gaddha Chowki in Kanchanpur District in far-western region; these posts aided in the early identification and tracking of imported cases. Given sustained malaria importation, the NMCP is once again considering year-round border screening as a surveillance approach for MMPs, yet plans have been delayed due to infrastructure challenges and questions around cost-effectiveness. More generally, the priority that the NMCP should place on reducing importation is unclear due to the lack of evidence of the extent to which imported infections may actually sustain indigenous transmission.

Epidemiological and mathematical modelling studies in numerous eliminating countries bordering higher burden neighbours demonstrate that transmission largely persists due to the continued pressure of imported cases [10–14]. Churcher et al. [12] provide statistical evidence that controlled non-endemic malaria (where the average number of new persons infected by a person with malaria is less than one) has been reached once the proportion of imported cases among detected cases reaches between 32 and 48%, depending on case burden.

Given the low malaria burden in Nepal and high cross-border movement in remaining endemic areas, there is a need to improve understanding of the subgroups of MMPs at greatest risk of importing malaria, their role in sustaining local transmission, and how to access them. There is currently limited information regarding basic characteristics of MMPs that may be necessary to design effective screening and prevention interventions, including operational questions of how they should be timed and targeted, such as the travel patterns of MMPs and the specific conditions that may lead to exposure during travel and while living and working abroad. Potential operational challenges that may limit access of MMPs to routine malaria prevention and treatment and pose a constraint to improving diagnosis and treatment have not been investigated [15, 16].

Relatedly, it is essential to understand whether current surveillance approaches effectively detect cases and accurately assess levels of malaria prevalence among MMPs at the population level. Due to frequent travel, MMPs may be under-represented in routine case data and absent during household visits. Facility-based surveillance approaches may also fail to capture MMPs who face barriers to accessing public health facilities, prefer private facilities, or who do not seek care at all [17, 18]. While several complementary, targeted surveillance strategies are increasingly under investigation for reaching subgroups at elevated risk for malaria, such as border screening, respondent-driven sampling (RDS) and time-location sampling (TLS) [19, 20], it is unclear whether these approaches would be appropriate for high-risk MMPs in Nepal.

This formative research study aimed to address these knowledge gaps in far and mid-western Nepal, specifically to (1) quantify the association between imported and local transmission, (2) document the social determinants of imported malaria, (3) characterize high-risk MMPs, and (4) identify potential surveillance and intervention strategies in MMPs and how they may be optimized through spatial and temporal targeting.

## Methods

### Study design

This study employed a mixed-methods approach, including (i) a retrospective analysis of passive surveillance data to define temporal and spatial relationships between imported and indigenous malaria cases, (ii) a quantitative health facility-based survey of imported cases and their MMP social contacts to define characteristics and travel patterns of this population, and (iii) focus group (FG) discussions and key informant interviews (KIIs) to explore opportunities and barriers for targeted malaria surveillance in MMPs. Data collection was carried out over a 3-month period between August and November 2016.

### Study sites

**Fig. 1:**
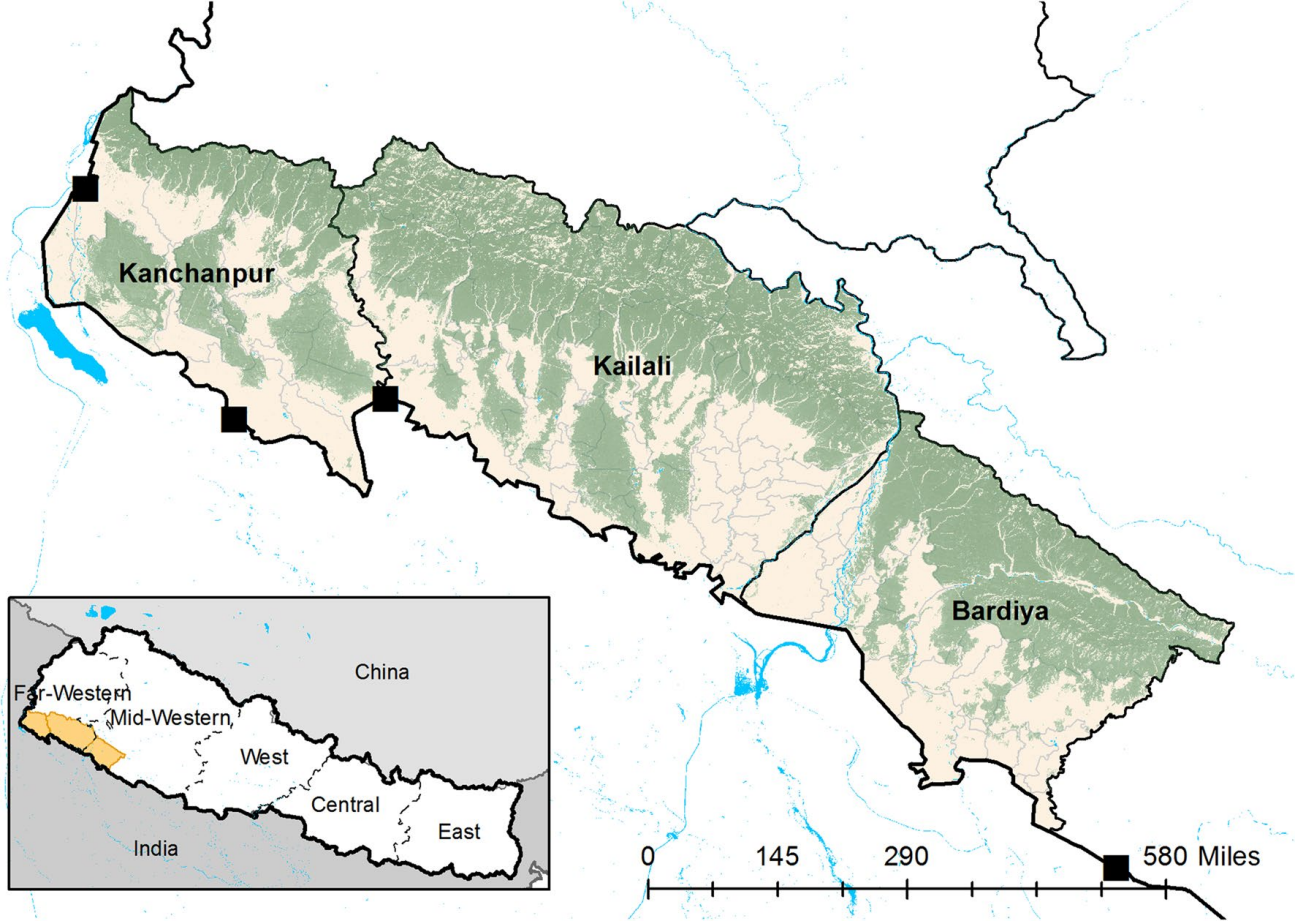
Study site districts (Kanchanpur, Kailali and Bardiya) and formal border crossings (black squares) in far-western and mid-western Nepal (inset). Green shaded areas represent tree cover

The study was conducted in three historically high-burden border districts in far and mid-western Nepal with high population movement to India: Kanchanpur, Kailali and Bardiya (Fig. 1). These districts form part of the lowland *terai* in western Nepal, where malaria transmission is concentrated, and key foci bordering the malaria-endemic, Indian states of Uttar Pradesh and Uttarakhand [5]. *Plasmodium vivax* predominates malaria transmission, which is highly seasonal with the majority of transmission occurring from June to September during the monsoon season [9]. The primary malaria vectors are *Anopheles fluviatilis* and *Anopheles maculatus* (responsible for transmission in the forest fringe, foothills and inner terai) and *Anopheles annularis*, an inefficient vector found in the outer *terai*. Historically, *Anopheles minimus* was also an important and highly efficient vector in forest and forest-fringe areas, but was reported to be eliminated following DDT spraying [9].

Together, the three study districts have a combined population of 1.9 million and accounted for 34% of the total burden of malaria in Nepal in 2016, with the annual parasite incidence (API) ranging from 0.27 cases per 1000 population in Kailali to 0.14 and 0.07 in Kanchanpur and Bardiya [21]. There is a large private health sector in these districts, but private clinics and pharmacies remain largely unregulated and have low case reporting rates. Based on data from the 2014 Malaria Indicator Cluster Survey, febrile children under the age of 5 years in the far western *terai* were more likely to seek treatment with a private provider (49.0%) than at a public health facility (26.4%) [22]. An estimated 0.7 million migrants cross annually at four formal border check points on the border with India [23]. Planned implementation of a border malaria check post at the main crossing in Kanchanpur district (Gaddha Chowki) has been delayed since 2013 due to resource limitations [9].

### Case definitions and surveillance strategies considered

Following WHO and Nepal national guidelines, all study components defined a malaria case as a person in which the presence of malaria parasites has been confirmed by microscopy or rapid diagnostic test (RDT) [24]. All *P. vivax* cases were assumed to be incident, based on a lack of identifying information to distinguish potentially relapsing infections. Imported cases were defined according to national guidelines as malaria cases confirmed who moved from their permanent or previous residence within the past 4 weeks to stay or work in a different district or country [25]. This working definition was revised during the pilot stage of prospective data collection due to low case numbers and the predominance of *P. vivax*. The revised definition extended the period of exposure to include overnight travel within the prior 7–60 days to a destination outside of Nepal or to a “high-risk” district, defined as districts in which indigenous cases have been reported in the past 3 years.

Three possible active malaria surveillance strategies were assessed during the qualitative portion of this study. These approaches are currently or have been under consideration in Nepal to identify and access MMPs for malaria screening and treatment.

- *Border screening* Border screening is the targeted screening of cross-border travellers at or near a border crossing and has had limited use in Nepal. Historically, this approach has had some success in Nepal but is generally implemented either through enforced or voluntary testing, and has had mixed success due to low case yield, limited acceptability and high operational costs [26].
- *Peer*-*referral recruitment* Peer-referral recruitment is a technique for accessing specific populations where individuals (in this case imported malaria cases) refer acquaintances who meet specified characteristics (e.g. cross-border travel or behaviours that may lead to exposure). Peer-referral can be used to improve the coverage of outreach, education and prevention. The approach can also be applied to case detection, in addition to standard active case detection (ACD) to identify peers at high risk of infection. Peer-referral strategies to support malaria elimination are currently under investigation in several contexts [20].
- *Venue*-*based recruitment* Venue-based recruitment is a technique to access members of a high risk-population at specific venues or locations (i.e. parks, tea shops, bus stops) where they are known to gather. This method, too, is often used for purposes of outreach, prevention and as a sampling method for surveys [27].

### Retrospective study

#### Study design and population

This was a retrospective analysis using passively detected case-based surveillance data from Kanchanpur, Kailali and Bardiya. In Nepal, confirmed malaria case data are routinely entered into patient registers at all public and private health posts. These data are then reported to the district health office, which collates the information and submits centrally to the Health Management Information System (HMIS). In this study, de-identified case-level data from the three study districts were extracted from the HMIS for the period January 2013 to December 2016.

#### Data collection and management

Extracted variables included date of diagnosis, age, gender, travel destination (imported cases only), location diagnosed, location of residence, diagnostic test (RDT or microscopy), malaria speciation (if available) and whether the individual was treated. Data were entered into a standardized excel database and linked to the corresponding village development committee (VDC) or municipality (MC) of residence for mapping.

#### Data analysis

Retrospective case data across the three districts were summarised according to species, month and importation status. Monthly imported and indigenous case numbers were aggregated by village development committee/municipality (VDC/MC) and used to calculate monthly incidence rates using population denominators provided by the NMCP. These data are estimated from the 2011 National Population and Housing Census in Nepal and assume a 1.35 annual growth rate [28]. Annual incidence of imported and indigenous cases were mapped using ArcGIS 10.4 (Redlands, CA) to visualize variation in transmission across the three districts and time period.

The association between monthly indigenous case counts (outcome) and importation rates in the previous month was investigated using Poisson regression models in ‘R-INLA’, which is a widely used software library for approximating Bayesian inference. A non-spatial Poisson regression model was used to initially test univariate associations of importation rates and potential environmental risk factors with monthly indigenous case counts (see Additional file 1). Environmental covariates with a *P* value of < 0.05 were included in the initial multivariate model with logged importation rate, district and year as fixed effects and evaluated using a process of backwards-stepwise elimination. Covariates were retained where the deviance information criteria (DIC) was lower or changed the primary association of interest more than 10%. Additional details of this covariate selection process are described in Additional file 1, which includes a list of all potential environmental factors, spatiotemporal resolutions and sources and a summary of their association with malaria incidence. The software version used for this study was *R* version 3.4.

All environmental covariates selected via the preliminary analysis, importation rates in the previous month, reported cases and catchment population were included in a Bayesian spatio-temporal model. Different models were tested and compared using leave-one-out cross-validation based on the conditional predictive ordinate (CPO). The best fit model was a conditional autoregressive (CAR) Poisson model with a periodicity of 12 months, which captures the month-to-month changes in transmission as well as seasonal trends. The details of the model selection process and comparison of candidate models are provided in Additional file 2.

### Cross-sectional survey

#### Study design and population

A cross-sectional survey was conducted amongst imported index cases and a sample of MMPs, selected using snowball sampling. All malaria cases diagnosed at health facilities in the three districts between August 31 and November 15, 2016, were screened according to eligibility criteria in Table 1 and entered into a recruitment registry by health facility staff. Imported cases were asked to provide information for up to five MMP social contacts who met specified eligibility criteria (Table 1), including travel in the prior 60 days. Research assistants then screened MMP social contacts by phone to confirm eligibility and invited eligible individuals to participate in the study.

**Table 1:** Eligibility criteria for imported cases, MMP social contacts and locally acquired malaria cases included in prospective surveys and/or focus group (FG)

Imported case *(survey and FG)*	MMP social contact *(survey and FG)*^a^	Locally acquired malaria case *(FG only)*
Confirmed malaria infection by microscopy and/or RDT Aged over 18 years Can speak and understand Nepali First-time participant in the study Spent at least one night outside of study district in the past 7–60 days, either outside of Nepal or in a specified high-risk district in Nepal	Aged over 18 years Can speak and understand Nepali First-time participant in the study Spent at least one night outside of study district in the past 60 days, in the same destination as referring case	Confirmed malaria infection by microscopy and/or RDT Aged over 18 years Can speak and understand Nepali First-time participant in the study Has not spent the night outside of study district in the past 7–60 days

^a^At most, only one social contact may be a member of the referring cases’ household

As the cross-sectional survey was conceived of as a pilot study, no formal sample size calculation was conducted.

#### Data collection and management

Informed consent was obtained by research assistants or a trained health facility staff member prior to administration of a structured questionnaire, which collected information on socio-demographics, occupation, travel histories, malaria prevention and outdoor exposures at night.

MMP social contacts answered additional questions around history of malaria and treatment seeking. All questionnaire data were entered from paper forms into an electronic database using ODK Collect, and regularly uploaded to a central server.

#### Data analysis

Quantitative data from structured interviews were summarised to develop profiles of imported cases and MMP social contacts. Specific areas of focus included socio-demographic characteristics, travel patterns and malaria attitudes, knowledge and practices. Categorical variables were tabulated and categories combined where levels contained < 5% of responses.

Of 138 specific travel destinations in India, all locations were geolocated to a specific longitude and latitude corresponding to the cited village/city (87%) or greater municipality (13%) using a number of electronic sources of information, including GeoNet Names Server [29], Google Earth [30] and Falling Grain [31]. Similarly, the majority of named transit points (238/246) were successfully geolocated. Locations identified from one source were cross-checked against other sources and to ensure that they fell within the correct administrative area or municipality. Travel routes were condensed and maps created in ArcGIS.

MMP social contacts provided a statistical comparison to cases to identify risk factors for malaria infection within MMPs. Conditional logistic regression was carried out in STATA v14 (College Station, Texas) to identify potential risk factors that may distinguish MMPs known to be infected with malaria (imported cases) from their peers. Age and gender were adjusted for a priori as confounders, and key exposures assessed through this approach included occupation, type of work (indoor *vs* outdoor), duration of travel, transit times and destination.

### Focus group discussions and key informant interviews

#### Study design and population

Focus groups (FGs) were conducted with a convenience sample of eligible indigenous cases, imported cases, and MMP social contacts. Nine FGs were conducted in-person with 56 participants in two districts, Kailali and Kanchanpur. This included seven FGs with 43 imported cases and MMP referrals in Kailali (4) and Kanchanpur (3) and one FG consisting of 6–7 indigenous cases in each district. A minimum of six FGs is usually considered sufficient to achieve data saturation in a group and allowed us some comparison between MMP and local populations.

Ten key informants were selected in each district by the study coordinator based on their perceived knowledge of MMPs in this area of Nepal. Participants were generally senior-level employees and represented a broad range of individuals with knowledge about MMPs, including non-governmental organizations (NGOs), health officials, political parties, representatives of hotel associations and police/immigration officials.

#### Data collection and management

FGs and key informant interviews were scheduled through mobile phone communication and the response rate was 100%. FGs and key informant interviews were conducted and transcribed in the Nepali language by a Nepalese research team consisting of a moderator and note-taker. The moderator facilitated discussions based on a thematic guide and sessions were audio recorded with detailed notes captured by the note-taker. All participants were oriented to the study and provided a brief description of why the research was being conducted, prior to being administered written informed consent. FGs were conducted at health facilities in Nepali and lasted around 1 h. Key informant interviews were conducted at the participants’ workplaces.

All FGs and interviews were audio recorded. The research team carried out a debriefing session immediately after completion of the session and a summary form was completed based on the discussion and notes. Thematic guides were pilot tested and revised before the start of data collection. Themes explored in the FGs and in-depth KIIs included travel and behavioural exposures related to malaria, health-seeking behaviours and perceptions of proposed active surveillance and response strategies.

#### Data analysis

FGs and KIIs were transcribed from audio recordings within 10–30 days. Nepali transcripts were then translated into English and formally analysed using a thematic content analysis approach. Specifically, transcripts were read and an analytic codebook developed around the main themes in the topic guides. Codes and transcripts were organized and analysed in Dedoose [32], including coding segments of textual data and grouping them into discrete units. Additional categories emerging from the data were added to the codebook as necessary and the final codebook was applied by two independent coders, following training to reach a high level of concordance. Themes uniting the categories were identified and relations between and within codes were used to provide additional insight into the categories and qualitative aims of this study.

Thematic analysis identified four main areas of discovery:

1. Experience with malaria and risk perception (locally and while travelling).
2. Mobility and travel patterns.
3. Current treatment-seeking and preventive behaviours.
4. Opportunities and barriers to accessing MMPs through specific active surveillance strategies.

Results for MMPs were qualitatively compared to indigenous cases to identify any key differences. Qualitative information was used to add detail and perspective to our understanding of the factors that influence treatment seeking and travel behaviours, as well as identify opportunities and barriers to specific active surveillance approaches in MMPs.

## Results

### Retrospective analysis

There were 1640 individual malaria case records reported through passive health facility based surveillance data from the three districts between 2013 and 2016, including 1418 cases of *P. vivax*, 184 cases of *Plasmodium falciparum*, and 38 mixed infections. Data were geo-located to the VDC of residence for the majority (95.8%) of records, and a comparison with unpublished district-level data (health facility based HMIS reporting monthly to the district public health office) showed good correspondence in Bardiya and Kanchanpur, but 24–35% under-reporting in Kailali (see Additional file 3) [12].

Overall, approximately half (54.1%) of malaria cases diagnosed between 2013 and 2016 were classified as imported by district health officers based on their travel history. Both indigenous and imported cases were typically adult males (Table 2), and imported case profiles increasingly aligned with this demographic group over this time period.

**Table 2:** Demographic and clinical characteristics of 1640 locally acquired and imported cases from 2013 to 2016

	Local (N = 753)		Imported (N = 887)		P value
	n	(%)	n	(%)	
Male	560	(74.4)	756	(85.2)	< 0.0001
Age category (years)					
< 5	17	(2.3)	10	(1.1)	0.06
5–14	56	(7.4)	49	(5.5)	
15–29	363	(48.2)	464	(52.3)	
30–59	267	(35.5)	320	(36.1)	
60+	50	(6.6)	44	(5.0)	
Plasmodium species					
*P. falciparum*	103	(13.7)	81	(9.1)	< 0.0001
*P. vivax*	620	(82.3)	798	(90.0)	
Mixed	30	(4.0)	8	(0.9)	
Diagnostic					
RDT	285	(37.9)	250	(28.2)	< 0.0001
Microscopy	444	(59.0)	575	(64.8)	
Both	24	(3.2)	62	(6.7)	
District					
Kailali	498	(66.1)	567	(63.9)	0.244
Kanchanpur	183	(24.3)	246	(27.7)	
Bardiya	72	(9.6)	74	(8.3)	
Year					
2013	338	(44.9)	290	(32.7)	< 0.0001
2014	192	(25.5)	265	(29.9)	
2015	94	(12.5)	185	(20.9)	
2016	129	(17.1)	147	(16.6)	

#### Temporal and spatial trends in transmission

**Fig. 2:**
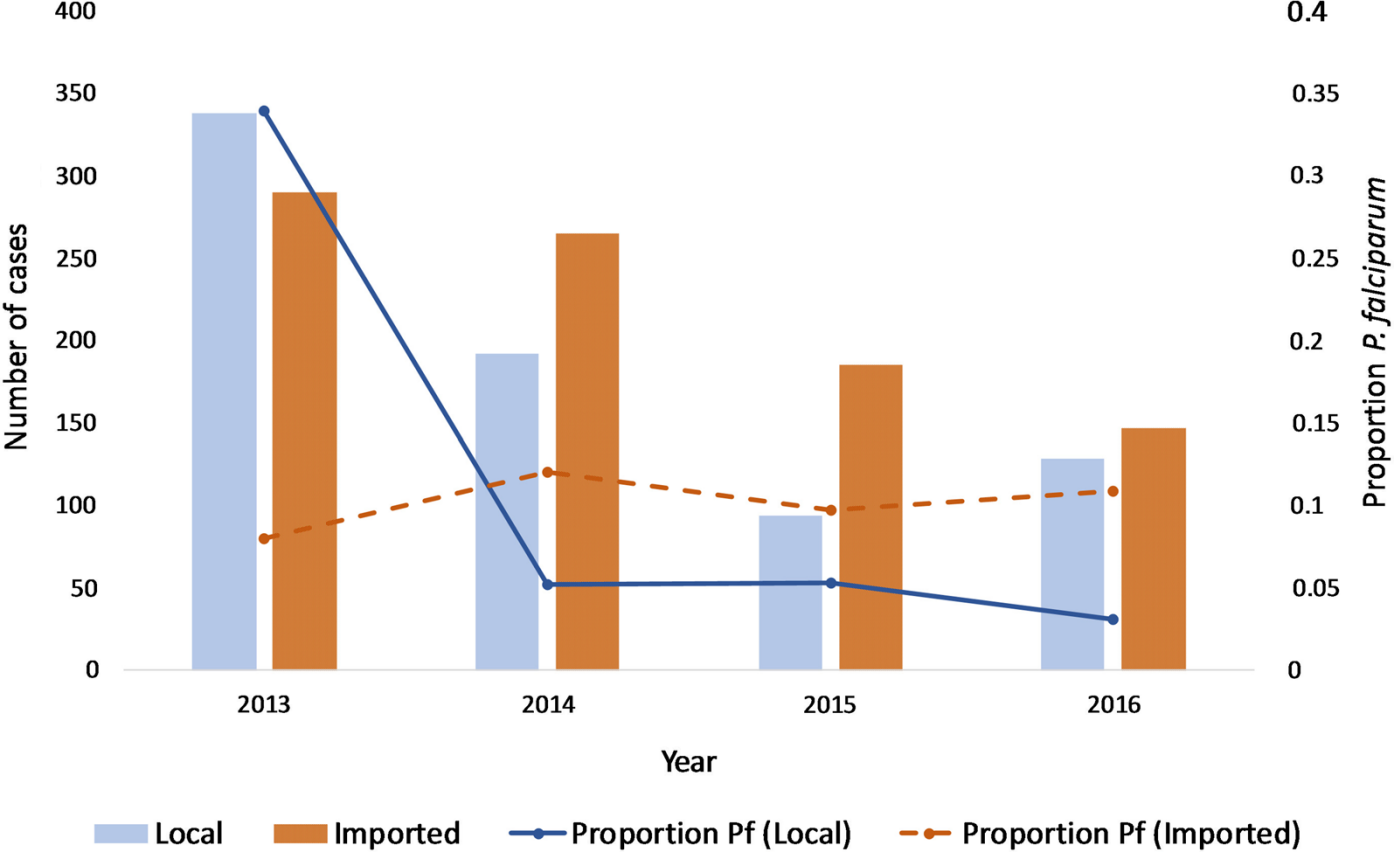
Total number of indigenous and imported cases (bars) and respective proportion of *Plasmodium falciparum* (Pf) cases (lines) in the three study districts between 2013 and 2016. While the number of indigenous falciparum cases fell in 2014, corresponding with a spike in the use of RDTs, the proportion of imported falciparum cases was stable

The number of imported cases dropped from 290 to 147 and indigenous cases from 338 to 129 between 2013 and 2016; corresponding to an increase in the proportion of imported cases (46% to 59.8%) (Table 2). Both imported and indigenous malaria transmission in this area of Nepal continues to be dominated by *P. vivax*, although there has been an overall decline in cases during this period (Fig. 2). In 2014, the number of *P. falciparum* cases classified as indigenous dropped by 91% (from 88 to 8), which corresponded with a spike in use of RDTs. Although only 10% of imported cases were infected with *P. falciparum* in 2016, these were the majority (77.8%) of all diagnosed *P. falciparum* cases.

Imported cases had distinct bimodal seasonality, with the first peak occurring in April-June and a second peak in August–September (see Additional file 2: Fig. S2). These periods roughly correspond to when travellers commonly return back to Nepal for a month for agricultural activities (harvesting wheat and paddy cultivation) during April–June and return for festivals and harvesting of rice, other grains, and paddies in September–October. In contrast to imported malaria, indigenous malaria transmission in Nepal showed a single, extended peak and greater variation between years (see in Additional file 2: Fig. S2).

**Fig. 3:**
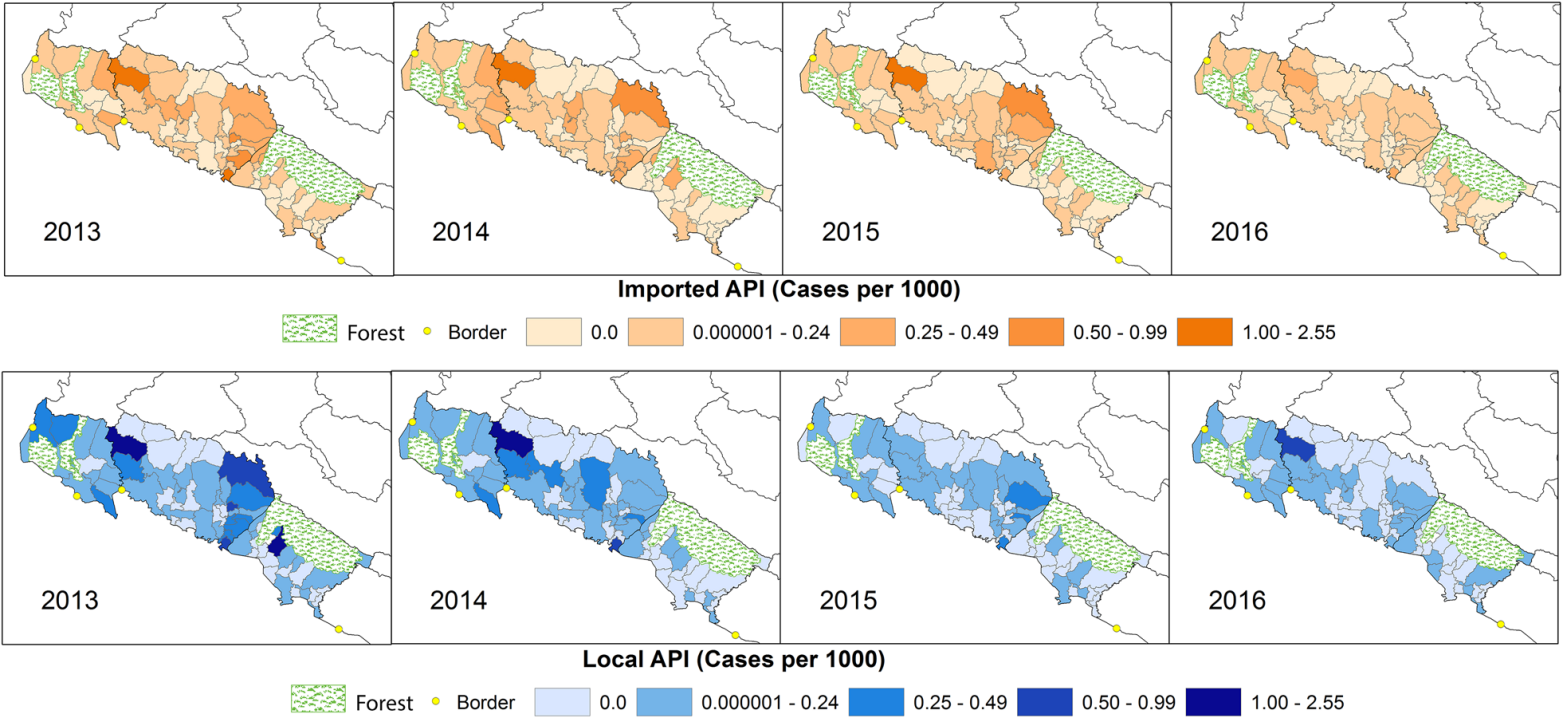
All species imported and indigenous annual parasite index (API) at the village development committee (VDC) level in the three study districts between 2013 and 2016. Areas of higher transmission are located in the south of Kailali bordering Bardiya National Park and on the northern border with Kanchanpur. VDCs that are predominantly covered by a national forest with very low populations are shaded green

**Fig. 4:**
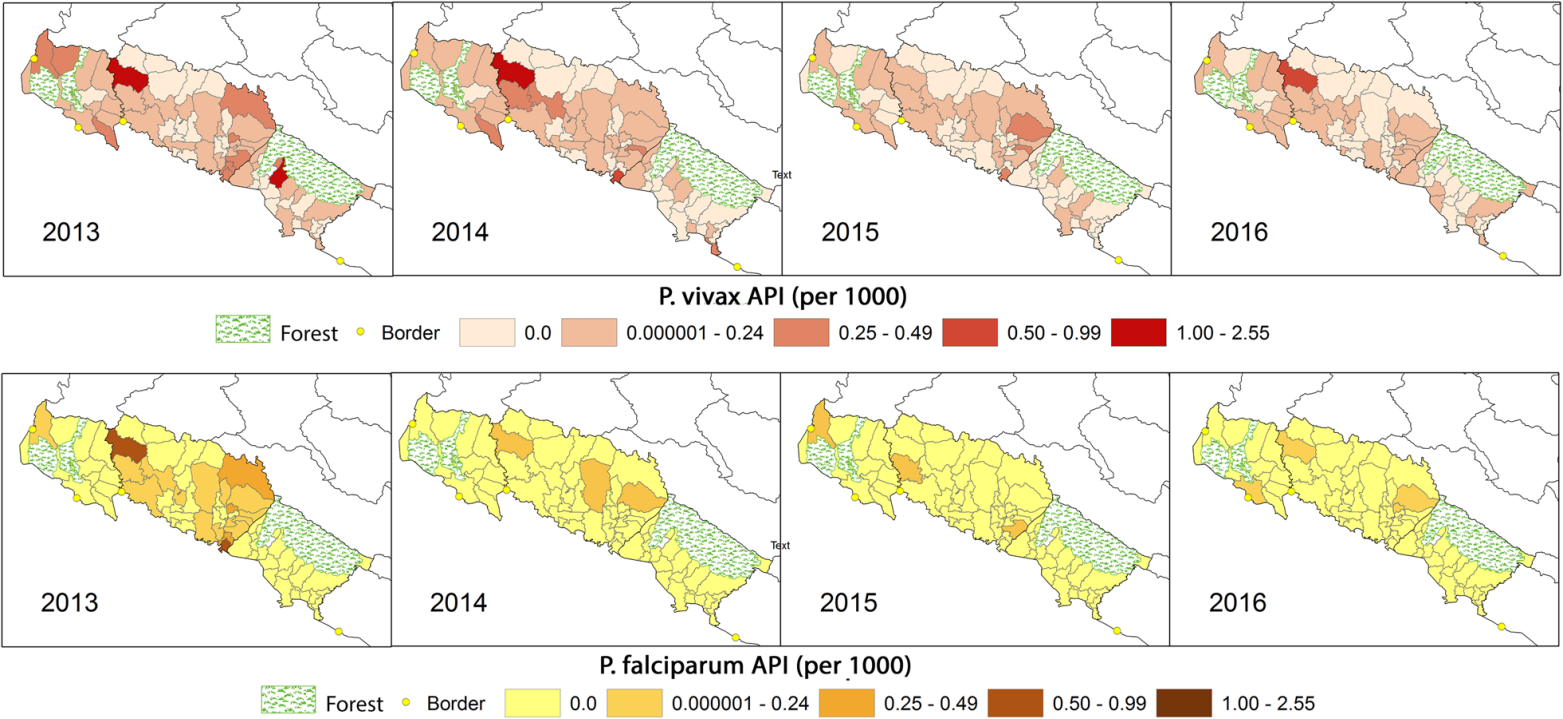
Indigenous annual parasite index (API) of *Plasmodium vivax* and *P. falciparum* at the village development committee (VDC) level in the three study districts between 2013 and 2016. National forests are shaded green

The geographic distribution of imported and indigenous malaria cases between 2013 and 2016 overlapped and foci were predominately observed in northern Kailali, crossing into Kanchanpur (including Godawari VDC and bordering VDCs), as well as southern VDCs in Kailali that border Bardiya (Fig. 3). Foci tended to occur in areas of low to mid population density and the decrease in incidence over time corresponded with smaller geographic foci of indigenous transmission. Species-specific maps of indigenous annual parasite incidence (API) for these years showed similar trends, but highlighted the precipitous decline and highly focal distribution of *P. falciparum* in comparison to *P. vivax* in these areas (Fig. 4).

#### The role of imported malaria in local transmission

The number of indigenous malaria cases within a given VDC was directly and strongly related to higher levels of imported infections in the previous month, after adjusting for variation in rainfall, population density and year (Table 3). Each percent increase in the previous month’s importation rate was associated with a two percent increase in the risk of an indigenous infection (IRR 1.02 95% CI 1.01–1.03). After accounting for the effect of environmental covariates, Godawari and Dhansinghapur VDCs in Kailali remained at relatively high risk for unexplained reasons.

**Table 3:** Factors associated with monthly indigenous malaria incidence at the VDC level, January 2013–December 2016, in Kailali, Kanchanpur and Bardiya districts

	Posterior mean, (95% Crl)		
	All species	*P. vivax*	*P. falciparum*
Fixed effects (IRR)			
Lagged importation rate^a^	1.02 (1.009, 1.031)	1.025 (1.013, 1.037)	1.011 (0.962, 1.057)
LST (°C)	1.016 (0.978, 1.049)	1.007 (0.962, 1.05)	0.924 (0.811, 1.074)
Rainfall (mm)			
< 20	1		
20–119	1.117 (0.757, 1.618)	1.064 (0.684, 1.626)	0.124 (0.014, 1.633)
120–1450	1.334 (0.8, 2.065)	1.145 (0.642, 1.943)	0.198 (0.022, 2.642)
High population density^b^	0.614 (0.399, 0.947)	0.616 (0.398, 0.96)	0.579 (0.293, 1.233)
Year			
2013	1		
2014	0.635 (0.48, 0.838)	0.921 (0.674, 1.256)	0.072 (0.03, 0.156)
2015	0.442 (0.305, 0.64)	0.609 (0.408, 0.91)	0.07 (0.024, 0.174)
2016	0.636 (0.436, 0.924)	0.858 (0.568, 1.289)	0.09 (0.028, 0.241)
District			
Kailali			
Kanchanpur	0.475 (0.308, 0.736)	0.603 (0.389, 0.94)	0.024 (0.006, 0.078)
Bardiya	0.768 (0.486, 1.205)	1.035 (0.65, 1.634)	0 (0, 887.532)
Random effects (precision)			
Seasonal random effect	2.949 (0.052, 17.118)	1.017 (0.016, 6.351)	0.009 (0, 0.049)
VDC effect unstructured	2.25 (1.464, 3.329)	1.972 (1.295, 2.905)	3.527 (0.887, 10.115)
VDC effect structured	3013.549 (745.865, 7326.227)	2931.545 (708.639, 7408.301)	3415.982 (835.229, 9202.779)

Crl, Bayesian credible interval; C, celsius; mm, millimeter; IRR, incidence rate ratio; LST, land surface temperature; VDC, village development committee; DIC, deviance information criteria; CPO, conditional predictive ordinate

^a^Monthly cases per 1000 population, ^b^ defined as the top quartile

In species-specific models, the positive relationship between indigenous cases and lagged imported cases was similar, however only reached statistical significance in the *P. vivax* model (Table 3), potentially due to the low number of indigenous *P. falciparum* cases (N = 103), the majority of which were in 2013 (N = 88).

### Prospective studies

In total, 60 (98%) imported cases were recruited into the study from 19 health facilities between August 31 and November 15, 2016. Thirty-three (55%) imported cases provided contact details for at least one individual who had travelled outside of the district within the past 60 days, with a total of 100 MMP social contacts referred. Eighty (80%) of these MMP social contacts were successfully contacted and interviewed, reflecting successful recruitment from 27 imported cases. Imported cases who referred at least one contact had information for three individuals on average (ranging from 1 to 5) whom they believed met the criteria for inclusion. The vast majority of MMP contacts were either friends (50%) or family members (48%).

Characteristics of imported cases were similar to their MMP social contacts in terms of residency, gender, age distribution and education level, supporting the theory that they are part of the same source population (Table 4). Amongst imported cases, individuals diagnosed with *P. vivax* were more likely to be male and reside in Kailali compared to *P. falciparum*.

**Table 4:** Profiles of imported cases and MMP social contacts from three districts, September–November 2016

Key characteristics	Imported cases							MMP social contacts		P value^b^
	Pf		Pv		P value^a^	Total		N = 80	(%)	
	N = 11	(%)	N = 49	(%)		N = 60	(%)			
District of diagnosis										
Bardiya	4	(36)	3	(6)	*0.01*	7	(12)	2	(2)	0.07
Kailali	4	(36)	34	(69)		38	(63)	51	(64)	
Kanchanpur	3	(27)	12	(25)		15	(25)	27	(34)	
District of residence										
Bardiya	3	(30)	3	(6)	0.22	6	(10)	2	(3)	0.06
Kailali	4	(40)	39	(61)		34	(58)	41	(51)	
Kanchanpur	3	(30)	14	(29)		17	(29)	37	(46)	
Dadeldhura/Baitadi	0	(0)	2	(4)		2	(4)	0	(0)	
Gender										
Male	9	(81)	48	(98)	*0.03*	57	(95)	71	(89)	0.19
Female	2	(18)	1	(2)		3	(5)	9	(11)	
Age category (years)										
15–30	6	(54)	26	(53)	0.99	32	(53)	49	(61)	0.42
30–45	4	(36)	19	(39)		23	(38)	28	(35)	
45+	1	(9)	4	(8)		5	(8)	3	(4)	
Education										
None	2	(18)	7	(14)	0.47	9	(15)	27	(34)	0.14
Primary	5	(46)	26	(53)		31	(52)	31	(39)	
Secondary	4	(36)	10	(20)		14	(23)	17	(21)	
Post sec.	0	(0.0)	6	(12)		6	(10)	5	(6)	

Italic values indicate significance of p value (p < 0.05)

Pf, *Plasmodium falciparum*; Pv, *Plasmodium vivax*

^a^Chi squared test of differences in key characteristics between Pf and Pv in imported cases

^b^Chi squared test of difference in key characteristics between imported cases and MMP social contacts

Summary statistics for FG participants are provided in Additional file 4. Ten KIIs were conducted in each of the three districts.

#### MMP travel profiles

Travel profiles of MMP cases and their social contacts show that the overwhelming majority had travelled to India (91.3%) for work during their last trip (85.0%) (Table 5 and Fig. 4). The two most common destinations were Surat (Gujarat, India) and Mumbai (Maharashtra, India), which made up nearly half (45.0%) of the most recent trips. Due to the respective distance and reason for travel, median transportation time averaged 3 days (IQR 1.5) and trips were of long duration (median of 5 months; IQR 9 months). While only a fifth of trips were reported as seasonal travel, many participants returned home to Nepal in September or October to celebrate festival season, which also coincides with the harvests, as described by the following quote, among others:

**Table 5:** Travel characteristics of 140 MMPs, including both imported cases and MMP social contacts

Summary trip characteristics	N = 150	(%)	Characteristics of most recent trip	N = 140	(%)
# trips reported in prior 6–60 days			Destination of travel		
1st trip	140	(93.3)	Surat, Gujarat, India	30	(21.4)
2nd trip	8	(5.3)	Mumbai, Maharashtra, India	33	(23.6)
3rd trip	2	(1.3)	Chennai, Tamil Nadu	12	(8.6)
Country of destination			Other, Gujarat, India	15	(10.7)
India	137	(91.3)	Other, India^a^	38	(27.1)
Congo, Africa	12	(8.0)	Congo, Africa	12	(8.6)
Nepal	1	(0.7)	Transit points^b^		
Frequency of travel to destination			Gaurifanta, Nepal	58	(41.4)
Once per year	70	(46.7)	Paliya, UP, India	85	(60.7)
> Once per year	18	(12.0)	Mumbai, India	32	(22.9)
< Once per year	60	(40.0)	Mathura, India	44	(31.4)
Don’t know/decline	2	(1.3)	Delhi, India	40	(28.6)
Median duration of travel (months) (IQR)	5	(9.0)	Worked at destination	119	(85.0)
Regular/seasonal travel to location	30	(20.0)	Type of work (n = 119)		
Return in Sept/Oct/Nov (n = 30)	23	(76.7)	Factory	23	(19.3)
Reason for travel			Security guard	18	(15.1)
Work	113	(75.3)	Logging/construction	15	(10.7)
Family/friends	17	(11.3)	Diamond work	13	(10.9)
Education/holiday	13	(8.7)	Hotel service	17	(14.3)
Decline to answer	7	(4.7)	Other	33	(22.0)
# people travelled with (mean/sd)	77	229	Work location (n = 119)		
0	15	(10.9)	Inside	53	(44.5)
1–4	44	(31.9)	Outside	32	(26.9)
5–14	53	(38.4)	Both	34	(28.6)
15–30	17	(12.3)	Mosquitos while working	107	(76.4)
30+	9	(6.5)	Mosquitos while sleeping	130	(92.9)
Median transit time (days) (IQR)	3	(1.5)	Residence environment		
Public transit	150	(100)	Town	112	(80.0)
Border crossed			Rural/forest	15	(10.7)
No border	10	(6.7)	Other	13	(9.3)
Gaurifanta, Kailali	74	(49.3)	Malaria protection during trip		
Basiha/Parasan	18	(12.0)	None	112	(80.0)
Banbasa/Gadda Chauki	16	(10.7)	ITN	28	(20.0)
Rupaidiya, Nepalganj	5	(3.3)	Repellant	16	(11.4)
Airport	12	(8.0)	Chemoprophylaxis^c^	12	(8.6)
Jamunaha/Khatima	4	(2.7)	Covering clothing	4	(2.9)
Kamalpur/Belauri	11	(7.3)	Other	2	(1.4)

^a^Includes locations in Rajasthan, Uttarakhand, Andhra Pradesh, and others in order of frequency

^b^Multiple transit points possible, those where are least 20% of participants transited are listed

^c^Drug used was Maploquine

*“For these destinations I have just described, there is no fixed time. I travel anytime. But we often travel back to harvest crop and to celebrate festival season in Nepal.”* (Male, Kailali, FG)

**Fig. 5:**
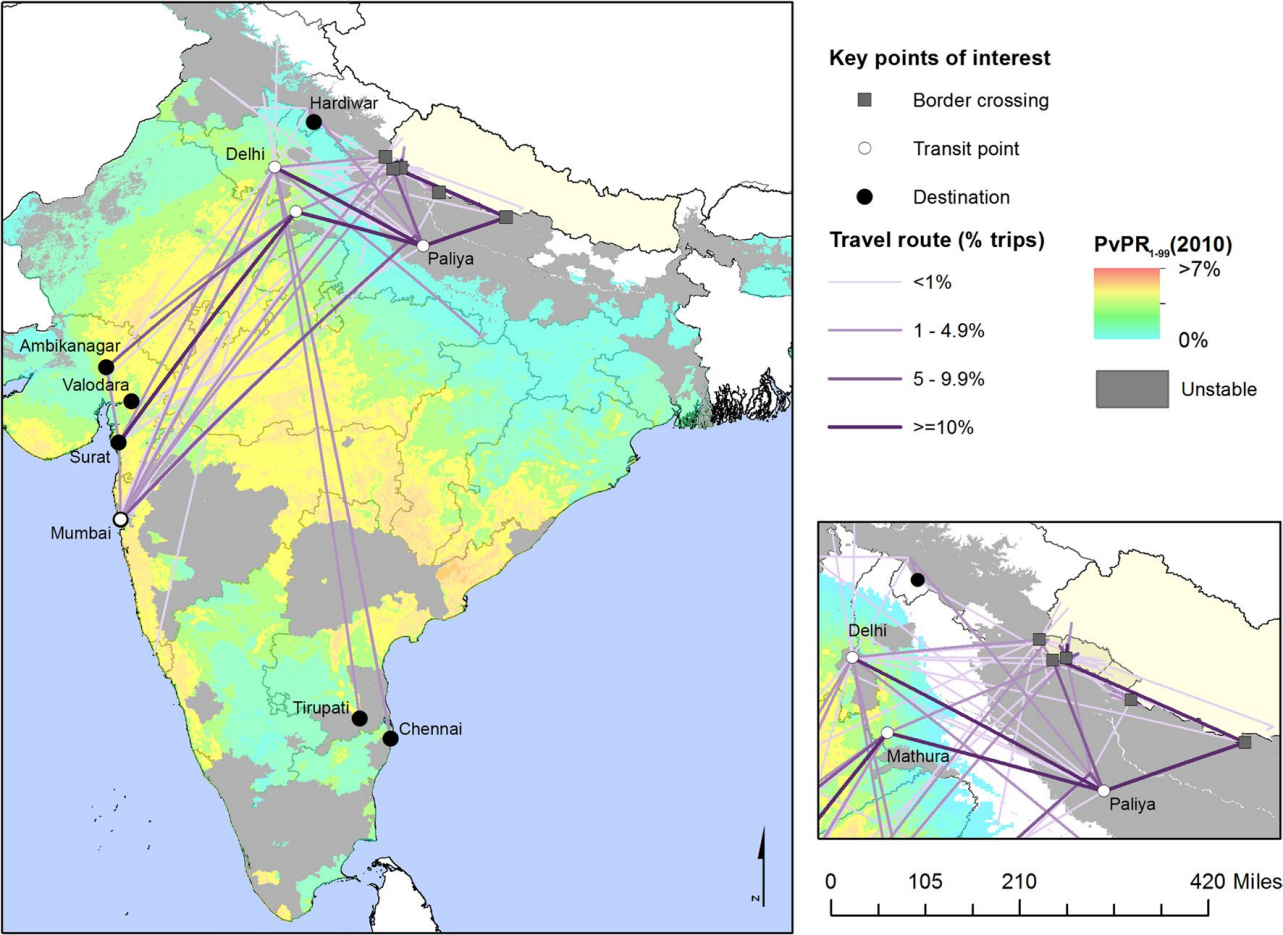
Destinations, travel routes and key transit points of 138 trips to India reported by 137 imported cases and MMP peers (Table 4), overlaid onto a map of predicted *Plasmodium vivax* all ages prevalence rate [44]

Characteristics that may suggest how to access MMPs for targeted surveillance include membership in specific occupational groups and common transit points along heavily trafficked travel routes. Common occupations included security guards (15.1%), factory work (19.3%), and hotel service (14.3%), but some forms of employment were specific to destination. For instance, Gujarat hosts more factory work (36%) while Maharashtra is common for security guard work (40%) or hotel jobs (24%). Travel routes and transit stops (Fig. 5) were similar among participants as all trips were taken via public bus or train.

#### Potential risk factors during travel

After adjusting for age and gender, MMP participants who worked outside had a sixfold increase in the odds of being an imported case compared with those who worked solely indoors (OR: 6.07, 95% CI 1.15–32.22). Working as a security guard, in particular, was perceived as a high-risk occupation by MMPs in FGs. While no specific destinations were associated with being a case rather than a contact in the survey data, longer transit times (in days) and shorter duration of travel (in months) were weakly associated with greater risk (OR: 13.9 95% CI 1.08–179.0 and OR: 0.74 95% CI 0.54–1.02) after adjusting for age, gender and working outdoors. FGs corroborated the potential risk of infection during travel, citing mosquitoes biting at transit points and within buses, as well as a higher perception of malaria risk at destinations further from Nepal (such as Gujarat or Mumbai).

#### Malaria knowledge and risk perception

Less than 50% of participants had heard of malaria when surveyed, and there were similar gaps in knowledge during FGs. In general, imported cases had better knowledge of malaria symptoms and availability of free testing compared to their MMP social contacts, which is likely due to their recent illness and subsequent diagnosis at a health facility (Table 6). Three quarters of MMP social contacts (75%) were unable to name one symptom of malaria; those who could were familiar with non-specific symptoms such as fever (20%) or headache (17%).

**Table 6:** MMP malaria knowledge, attitudes and practice and treatment seeking

	Imported cases		MMP social contacts		P-value^b^
	N = 60	(%)	N = 80	(%)	
Malaria knowledge, attitudes and practice					
Not heard of malaria	32	(53.3)	55	(68.8)	0.06
Knowledge of transmission					
Mosquito-borne	21	(35.0)	21	(26.3)	0.26
Don’t know	37	(61.7)	59	(73.8)	0.13
Other^a^	6	(8.3)	6	(3.8)	025
Knowledge of preventive measures					
0	40	(66.7)	58	(72.5)	0.22
1	7	(11.7)	13	(16.3)	
2+	13	(21.7)	9	(11.3)	
Type of preventive measures known					
ITNs	17	(28.3)	17	(21.3)	0.33
Chemoprophylaxis	10	(16.7)	8	(10.0)	0.24
Repellant	5	(8.3)	5	(6.3)	0.64
Covering clothing	3	(5.0)	5	(6.3)	0.76
Other	4	(6.67)	3	(3.75)	0.43
Knowledge of symptoms					
No symptoms known	29	(48.3)	60	(75.0)	*< 0.0001*
1–3 symptoms known	4	(6.7)	8	(10.0)	
4+ symptoms known	27	(45.0)	12	(15.0)	
Symptoms known					
Fever	31	(51.7)	20	(25.0)	*0.001*
Headache	25	(41.7)	17	(21.3)	*0.009*
Other (muscle pain, fatigue, dizzy)	29	(48.3)	12	(15.0)	*< 0.0001*
Knowledge of free testing and treatment					
Knows of free testing	28	(46.7)	18	(32.9)	*0.005*
Believes must pay	3	(5.0)	2	(2.5)	
Does not know	29	(48.3)	60	(75.0)	
History of symptoms and testing (MMP only)					
Ill with fever in past 6 months	–	–	16	(20.0)	–
Sought treatment for fever	–	–	16	(100)	–
Location sought treatment from	–	–			–
District hospital	–	–	1	(6.3)	–
Private clinic or hospital	–	–	5	(31.3)	–
Pharmacy	–	–	10	(62.5)	–
Diagnostic test used	–	–	15	(18.8)	–

Italic values indicate significance of p value (p < 0.05)

^a^Includes water-borne, food-borne and direct transmission

^b^Chi squared test of association

#### Health-seeking behaviours

There was a low awareness of free, government-provided malaria services at public facilities amongst MMPs (32.9%) (Table 6). Reported treatment-seeking behaviours at these locations were even lower: of the 20% (N = 16) of MMP social contacts reporting a fever in the prior 6 months, the vast majority (N = 15) sought treatment from the private health sector only (pharmacy or private clinic/hospital). Only one participant went to a public hospital for diagnosis. Qualitative findings from FGs and KIIs corroborated these findings and suggested that individuals would preferentially attend private facilities for fever and delay coming to the public health facilities until the fever is non-resolving or more severe. For example, one case stated:

*“The private clinic staff suggested that I go to the public health facility, since the government provides free services there. It was only after I had malaria several times that I went straight to the government health facility, because I knew.”* (Male, Kailali, FG)

Perceived barriers to treatment according to KIIs were lack of medicines at health posts and increased travel time. A number of key informants suggested that people may also seek treatment from traditional healers or travel to India for medical services, particularly at major transit points like Paliya.

#### Knowledge and use of malaria prevention methods

Knowledge of malaria prevention was low in MMP participants, with less than a third (21–28%) able to name ITNs as a method to protect against malaria infection (Table 6). Most travellers (80%) did not use any form of malaria prevention on their most recent trip, and ITN and repellant use were both low (20.0% and 11.4%) (Table 6). Despite this, many FG participants reported that they regularly used a net while at home in Nepal if available. While reported use of a net was common, KII and FG participants cited irregular distributions of nets, lack of knowledge around how and where to obtain nets, and low-income financial status as key barriers to ITN access in Nepal. Personal preference to not use the net due to the heat and perceived sensitivity to the chemicals in the net was also reported as a barrier to ITN use.

While travelling, lack of ITN use was generally attributed to lack of availability, affordability, and type of living quarters. Even if workers were in possession of a mosquito net while travelling, some participants would not be able to assemble it in their living quarters in India. A few participants commented on landlord restrictions around hanging nets in rented accommodation as a barrier to use:

*“We stay in rented house in India. House owner do not allow us to use nails striking on the wall, so it is impossible for us to use mosquito net. That is why we do not use mosquito net in India.”* (Male, Kailali, FG).

Some participants did mention burning wood or mosquito coils in India, however, and one participant reported that his living quarters in Surat were sprayed for mosquitoes.

#### Opportunities and barriers for malaria screening

Participants were generally positive about targeted malaria surveillance strategies. Two common themes emerged from discussions regarding screening approaches. First, high levels of social connectivity amongst MMPs provide an entry point for surveillance and interventions. Most participants preferred to travel to India within a group, in part due to safety. Corroborating data from Table 5, which shows that MMPs tended to travel in small or moderate sized groups of 2–15 people (70.3%), participants stated:

*“Some people have two to three people in a group while some have six to seven people in a group while travelling.”* (Male, Kanchanpur, FG)

*“We travel in group of ten, twelve, fifteen and sometime twenty of us travel together.”* (Male, Kailali, FG)

In India, Nepalese MMPs tend to stay well connected and frequently live together in large communities of varying size (upwards of 400–500 people), with sometimes as many as 20 people from a single village. Imported cases reported a median of 8 MMP social contacts known at the travel destination and engaged in the same activity during the past 60 days with some individuals (n = 7) indicating high numbers of contacts (50 or more) (Table 7). Another potential entry point for venue-based surveillance are Nepalese societies, which organize large cultural gatherings in many common destinations in India, typically on Sundays with several hundred people attending. Finally, there are highly networked MMP individuals (for example, involved in transportation or local leaders) who could be recruited to support peer-driven interventions.

**Table 7:** Size and recruitment of peer travel networks of 58 imported cases over 63 trips to India

	Mean (median) [range]	SD
Total number of peers in case’s “same destination, same activity” travel network and…^a^	16 (21) [0 100]	21
…Anticipated to be present in study district in next 2 weeks, and…	3 (0) [0 20]	4
…Aged over 18 years	2 (0) [0 20]	4
Number for whom contact details were provided^b^	1 (0) [0 5]	2

Two cases excluded with outliers (850) where travel was to Africa

^a^Number of people who live or work in the study district in Nepal, who had travelled to the same destination in India and engaged in the same activity in the past 60 days

^b^Meeting the above criteria, maximum of 5

Second, optimal timing of screening would correspond with Dashain and Tihar festivals, when people are most likely to return to Nepal for celebrations and agricultural harvesting.

*“There are seasonal migrants who come and go in seasonal period of time like during the agriculture season or for the major festivals. Like, they come during Ashadh, stay for a month then goes back again. After that they return in Ashoj for Dashian as well as to harvest the grain.”* (Male, Kailali, KII).

Barriers to malaria screening identified during FGs included unawareness of health services, particularly availability of free testing and treatment for malaria, in addition to low literacy, fear of medical visits, and the perception that screening is necessary only if one is ill. One FG referenced a recent polio immunization campaign implemented at the border, in which some people refused to participate based on the belief that they might be drugged or would feel faint.

#### Peer-referral recruitment

Strong social ties between Nepalese travellers offer an opportunity to conduct malaria screening through social networks. FG participants were confident that MMP social contacts could be recruited by mobile phone and would be willing to participate:

*“We have close friend, we can contact them, they will contact another friend and if we inform them, they will contact through mobile.”* (Male, Kailali, FG)

Most participants believed individuals referred by peers could be screened at their households or referred to health posts, schools or community meetings. There was variation in perceptions of when MMPs would most likely be free while in Nepal, with participants suggesting Sundays, weekday evenings after work, or mornings.

**Fig. 6:**
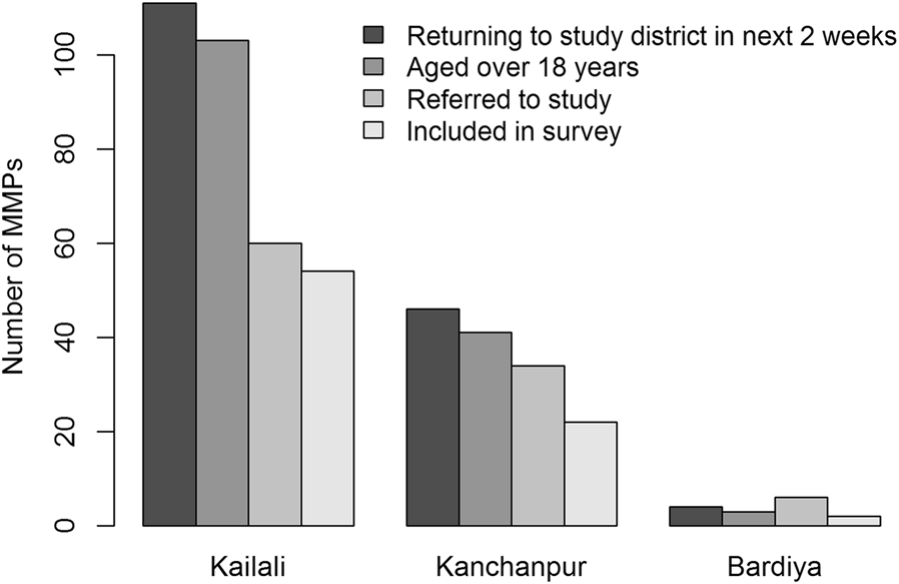
MMP peer network statistics of imported cases recruited in Kailali, Kanchanpur and Bardiya and reporting travel to India

In the context of this study, however, peer-referral resulted in enrollment of a fraction of the total MMP social contacts reported known by MMP cases involved in the same activity, in large part because few contacts were expected to return from India in the allotted two-week follow-up period (Table 7 and Fig. 6). Lack of contact details was another limitation. Overall, 1.3 (80/60) MMP contacts were successfully recruited per MMP case during the study, and 2.4 (80/33) MMP contacts were recruited, on average, for each of the MMP cases that provided any contact details.

#### Border screening

MMPs expressed mixed views of border screening and highlighted potential operational challenges. First, participants did not perceive any particular MMP subgroup to be at higher risk of fever upon return from India. All participants believed fevers are relatively rare and may appear before, during or after travel. Second, while survey findings indicated most MMPs travel through formal border crossings (Table 5), some FG participants mentioned some people (particularly those involved in illegal activities) may use informal border crossings. Some FG participants believe screening posts would be most successful if located at border stations or nearby roads or markets. Although some favoured mandatory, government-run screening to maximize participation, others cited barriers that included lack of time, concerns about safety at border check posts, and distrust of police:

*“When we travel from India then many police threaten us by asking money, asking our passport and citizenship, so people fear to participate in such gatherings. People fear if they will be asked money or get plundered in such programme.”* (Male, Kailali, FG)

#### Venue-based recruitment

Venues where MMPs tended to congregate in transit (in both Nepal and India) included tea shops, bus stations and rail stations. There were more specific locations identified in India, where Nepalese societies organize cultural events on Sundays and during festivals. Groups from a specific village may meet at specific markets in India or cinema halls.

*“Like in India, we have different Nepali community, if we contact there then it will be easy to spot all the people because Nepali people organize different cultural programme where most of the Nepalese attend, if you talk about this programme then it will be very easy.”* (Male, Kailali, FG)

Both Nepalese communities in Nepal and organized societies abroad have a leader or coordinator (President/VP) who are well networked through formal or informal channels. This person was identified as a point of contact to coordinate screening. Participants also suggested meeting at workplaces rather than recreational venues in India, and in particular, diamond factories in Surat, where they felt many Nepalese worked.

However, few potential venues were identified in Nepal and participants stated that individuals may have privacy concerns and refuse blood tests at public places identified, specifically tea shops or transit points. Differences in the timing of work shifts and availability are also likely to make venue-based recruitment in India more costly.

## Discussion

This study sought to address key knowledge gaps around malaria importation in far and mid-western Nepal, including investigating the association between imported and local transmission, better characterizing MMPs and identifying potential opportunities to optimize targeted surveillance and intervention strategies for this population. The analysis of retrospective case data demonstrated an association between (lagged) imported case rate and local rate in this area of Nepal, such that each percent increase in importation the month prior led to a two percent increase in local incidence rate. Recently imported cases and their MMP social contacts were found to be predominantly adult male labourers who travel to malaria endemic areas of India and were characterized by similar risk factors: they lacked a basic understanding of malaria transmission and prevention, rarely used ITNs while travelling, and when ill, tended not to seek treatment or prefer informal private providers. These findings suggest that many cases among these individuals may go undetected and that more targeted strategies are needed to improve coverage of education, testing, and surveillance in this population. To access high-risk MMPs more effectively, the study identified community-based surveillance approaches as the most promising options: strong social connectivity within these groups, concentration of Nepali MMPs in specific communities and workplaces within India; and well-attended seasonal festivals that motivate return to Nepal provide opportunities for peer-referral- and venue-based surveillance and interventions.

The finding that high-risk MMPs tend to be adult male labourers with a history of travel to India has been reported by previous studies in this area [3, 4] and is consistent with a profile of imported cases as adult males in other elimination contexts [2, 33]. The infrequent self-reported net use (20%) during travel contrasted with the high reported programmatic coverage (> 90%) of LLINs in Nepal since 2009 [34]. Even when home in Nepal, many of the MMPs interviewed were unsure how to obtain nets. MMPs may be less likely to receive nets during regular distributions in Nepal due to living in remote areas and absence from home during distributions. Innovative approaches are needed to make nets available to MMPs upon return from India, both for their own protection and to prevent onward transmission.

Given the high proportion of imported cases in this setting and receptivity of the *terai* region, some ongoing transmission is expected and will be mediated by the malaria species present and vectorial capacity. Past studies have shown that environmental variables are important drivers of vector density and seasonal patterns of malaria [35, 36], however the relationships, metrics and temporal lag periods used vary across settings and vectors [37]. This study demonstrates that receptivity in this area of Nepal is determined by higher levels of rainfall and temperature [38, 39]. Low use of public sector health services by MMPs is a key limitation of routine surveillance data and there is a need to incorporate private sector surveillance alongside campaigns to improve public sector use by these populations. In addition, improving the quality of routinely collected travel histories in case-based surveillance will allow automated analyses and mapping of sources and sinks on an ongoing basis to improve geographical targeting of interventions.

Accessing and achieving high coverage of vector control and surveillance interventions in MMPs is a key challenge to reducing malaria importation [19]. All three strategies examined (border screening, peer-referral, and targeting well-attended venues) were found to be feasible, however would need to be adapted to address the unique operational challenges identified. The finding of strong social connectivity among Nepalese travellers, while in transit and at destinations in India, suggests that peer-supported methods are promising to identify and contact MMPs to disseminate interventions. Improved knowledge of malaria risk avoidance, screening programmes, treatment options, ITNs, and repellants are shown to benefit travellers directly and improve access to and use of existing malaria health services [10].

Decades of research portray migration as a socially embedded process and support a role for utilizing existing social support and network structure to optimize behaviour change interventions [40, 41]. The findings from this study suggest that within Nepal, an obstacle to peer-driven methods is the delay until MMPs return home. This may be addressed by sending periodic follow-up reminders to refer MMP social contacts once they arrive, potentially by SMS as most MMPs were contactable by mobile phones. Peer promotors or navigators (MMP group members who are employed by a malaria programme to provide information or “navigate” health services) could also be employed to reach other MMPs while in transit and at destinations in India with high concentrations of Nepalese from far-western Nepal. Community leaders and established Nepalese societies were also identified that should be enlisted to promote uptake of peer-driven programmes or to directly provide screening or behaviour change communication (BCC) [42] to society members.

Beyond peer-driven approaches, we identified well-attended venues and work sites in India that could provide convenient physical locations to access large numbers of Nepali MMPs through a venue-based approach. Specific occupational groups working outdoors in malaria endemic destinations, such as security guards in Mumbai, could also be targeted upon return from India for voluntary screening. Border screening is resource and labour intensive, making it infeasible in many settings, but targeted approaches are likely to improve the test positivity rate and overall cost-effectiveness [43]. Study participants’ concerns about confidentiality of testing results and distrust of police, suggest that, just as in more stigmatized conditions such as HIV and STIs, malaria screening should be conducted in designated confidential areas within or nearby the venues and/or border check points and with careful selection of staff. Considering the high volume of border crossings during peak times, there is a clear need to use imported case data to construct high-risk profiles (potentially using a more representative MMP control group) to identify age, gender, travel and possibly occupational profiles to target screening to potentially asymptomatic individuals.

This study also found that longer transit times and shorter duration of stay abroad were weakly associated with a greater risk of malaria. Shorter-term migrants may perceive less risk, resulting in poorer malaria preventive behaviours, or have increased occupational exposures compared to longer-term migrants. Alternatively, imported cases may return to Nepal to seek treatment when they fall ill, which could result in a spurious association with shorter duration of travel. The qualitative portion of the study did not substantiate illness as a reason to return to Nepal.

Several methodological limitations should be considered. Radical cure of *P. vivax* is the standard of care in Nepal, but there is frequently low adherence to these guidelines from both the provider and patient populations. This is believed to be due to concerns around G6PD status and low compliance to the full 14-day course of medication. This study did not investigate these issues in MMPs, but it remains an important barrier to achieving elimination in Nepal. It was not possible to distinguish incident and potentially relapsing *P. vivax* infections in these analyses. Consequently, local and imported case numbers are likely to be overestimated and may obscure recent geographical trends. Case ascertainment was limited by lack of private sector involvement and, in the case of retrospective data, other reporting gaps. The prospective recruitment period was planned to coincide with the festival season but was a month delayed and subsequently short (3 months) in duration, which limited case and MMP recruitment and may affect generalizability. While a large proportion of imported cases (35%) and MMP contacts (33%) were interviewed on the same day as diagnosis or referral, the remainder were followed up within a week (75% and 39%) or longer. MMP contacts with follow up longer than 4 weeks (12.5%) were due to longer than expected return from travels. The data did not allow calculation of the total number of MMPs within the extended network or the maximum number of potential referrals, as there was no way of accounting for double-counting given the high degree of social connectivity and capped referrals at 5. However, it is likely that one round of peer-referral with a 2 weeks follow up will not be sufficient to recruit a large part of this network. Peer-referral recruitment was further limited by delays in enrollment, indicating that MMPs were not necessarily travelling back to Nepal in groups. In addition, MMP social contacts were sometimes lost to follow up due to incomplete contact information or residing in a remote area of the district, suggesting additional operational challenges for the peer-referral approach. Remote residence and scheduling conflicts were substantial challenges to organizing FGs, which may have influenced findings by restricting our sample to those with better access. The effect of this potential bias was minimized by reimbursing travel costs and working flexible hours to accommodate participants schedules and travel needs. Due to the clustered nature of the data (i.e. MMP social contacts were matched to the imported cases by destination) many potential risk factors are likely to be underestimated in the conditional logistic regression analysis. A more representative MMP control group, recruited through alternative means, would be useful in identifying additional risk factors to guide targeted screening approaches. Findings from the retrospective analysis should be interpreted with caution as it used area level data and observed correlation between imported and local cases could be introduced by regional seasonality or misclassification bias.

A cross-border strategy with India remains key to achieving and maintaining long-term elimination targets in Nepal. This study highlights the routes through which parasites move between Nepal and India and the surveillance gaps that make timely diagnosis and treatment in MMPs so challenging. Key informants also mentioned Indian migrants living in Nepal who may be vulnerable or contributing to local transmission and who are not represented in this study. Ensuring that MMPs from both sides of the border have adequate access to malaria prevention, testing and treatment along key routes and destinations will be critical to achieving regional elimination. A regional initiative in 2010 opened the door towards implementing cross-border activities for the control of three vector-borne diseases, including malaria [5]. Cross-border mechanisms to improve surveillance, information sharing and bilateral support are essential to reduce malaria transmission in MMPs and in highly trafficked destinations on both sides of the border, thereby benefiting both Nepalese and Indian populations.

## Conclusion

Population mobility and malaria importation from India are key challenges to malaria elimination in border areas of far and mid-western Nepal. This study has helped to better understand characteristics of MMPs and identify specific ways in which malaria surveillance and response can be adapted to optimize coverage and case detection in high-risk travellers. Specifically, intervention campaigns can tailor their timing, messaging and geographic focus to better target this population, and surveillance and response approaches can leverage strong social connectivity to improve intervention uptake. A combination of community and peer-referral based approaches will likely be needed to achieve malaria elimination in border areas.

## Additional files

**Additional file 1.** Covariate processing and selection methods.

**Additional file 2.** Model selection criteria and model comparison.

**Additional file 3.** Comparison of number of cases imported and total number of cases in 2016 reported through case-based surveillance and the World Malaria Report (WMR).

**Additional file 4.** Characteristics of focus group participants.
