# Supplementary material for: Designing malaria surveillance strategies for mobile and migrant populations in Nepal: a mixed-methods study
Source: Malar J. 2019 May 3;18:158. doi: 10.1186/s12936-019-2791-1 (PMC6500027; doi:10.1186/s12936-019-2791-1)
Supplement: Supplementary file 1 — Additional file 1. Covariate processing and selection methods. [file 12936_2019_2791_MOESM1_ESM.docx]

**Designing malaria surveillance strategies for mobile and migrant populations in Nepal: a mixed-methods study**

**Additional file 1. Covariate processing and selection methods**

Potential environmental covariates that could confound the association between monthly imported malaria infection rates (1 month lag) and indigenous incidence rates are summarized in Table A1.1. Mean digital elevation from Shuttle Radar Topography Mission and monthly climate indicators were derived at the VDC level from Climate Hazards Group Infra Red Precipitation with Station data (CHIRPS) (rainfall) and Moderate Resolution Imaging Spectroradiometer (MODIS) (enhanced vegetation index (EVI) and land surface temperature (LST))[20-22]. Static covariates included land cover data from MODIS, which was used to calculate the proportion of the VDC population living in areas classified as forest or woodland, as well as the proportion of the population living in areas classified as cropland. The mean population density per hector and distance in kilometers to a major road were calculated for each VDC/MC [23].

Non-spatial generalized linear regression models using a Poisson link function with population offset were implemented in the *stats* library in R and used to estimate univariate associations between all covariates and monthly indigenous incidence rates for species specific and all-species malaria. Estimated odds ratios and 95% confidence intervals are shown in Table 1.2, along with p-values from likelihood ratio tests against a null model. Environmental covariates with a p-value of <0.05 were included in the initial multivariate model with logged importation rate, district and year as fixed effects and evaluated using a process of backwards-stepwise elimination. Covariates were retained where the deviance information criteria (DIC) was lower or changed the primary association of interest more than 10%. Variance-inflation factors (VIF) were calculated for terms in each stepwise comparison to check for collinearity between environmental factors and to ensure all VIF values were below a threshold of 5.

Table A1.1 Potential environmental covariates for modelling indigenous malaria incidence with descriptions

| Covariate | Description | Units/scale | Spatial resolution | Temporal resolution | Source |
| --- | --- | --- | --- | --- | --- |
| Precipitation | Total precipitation in | Millimeters (mm) | ~5 km | Monthly | Climate Hazards Group InfraRed Precipitation with Station (CHIRPS) |
| EVI | Enhanced Vegetation Index | - | 1 km | 8 day composites averaged for month | Moderate Resolution Imaging Spectroradiometer (MODIS) |
| Temperature (day) | Mean land surface temperature (day) | Degrees Celsius | 1 km | 8 day composites averaged for month | Moderate Resolution Imaging Spectroradiometer (MODIS) |
| Proportion population in forest/woodland | Proportion of VDC (gridded) population residing in forest/woodlands | - | - | - | Derived from MODIS landcover data and Worldpop gridded population |
| Proportion population in cropland | Proportion of VDC (gridded) population residing in cropland | - | - | - | Derived from MODIS landcover data and Worldpop gridded population |
| Distance to road | Euclidean distance to road | Kilometers | 1 km | - | Derived using R and road shapefile |
| Population density | Population density calculated from population counts within each census unit. | People per hectare | ~100 m | Annual | Worldpop dataset  [www.worldpop.org] |
| Elevation | Average height above sea level | Meters | 90 m | - | Shuttle Radar Topography Mission [http://srtm.usgs.gov/] |

Table A1.2 Univariate associations between potential confounding environmental risk factors and monthly indigenous malaria case counts in the non-spatial, fixed-effects generalized linear regression model.

|  |  | ***Plasmodium vivax***  **N = 628^1^** | | ***Plasmodium falciparum***  **N = 123^1^** | | **All-species malaria**  **N = 727** | |
| --- | --- | --- | --- | --- | --- | --- | --- |
| **Covariate** |  | **OR (95% CI)** | **P-value** | **OR (95% CI)** | **P-value** | **OR (95% CI)** | **P-value** |
| Importation rate |  | 1.029 (1.027-1.031) | <0.0001 | 1.003 (0.993-1.011) | 0.598 | 1.027 (1.025-1.029) | <0.0001 |
| Rainfall (mm): | <20 | 1 | <0.0001 | 1 | <0.0001 | 1 | <0.0001 |
|  | 20-119 | 1.313 (1.226-1.406) |  | 1.077 (1.944-1.232) |  | 1.338 (1.257-1.425) |  |
|  | 120-1450 | 1.116 (1.040-1.198) |  | 0.429 (0.364-0.505) |  | 1.015 (0.950-1.084) |  |
| EVI: | <0.5 | 1 | <0.0001 | 1 | <0.0001 | 1 | <0.0001 |
|  | 0.5-0.9 | 1.178 (1.116-1.242) |  | 1.263 (1.110-1.434) |  | 1.152 (1.096-1.212) |  |
|  | 1.0+ | 1.030 (0.946-1.120) |  | 2.541 (2.196-2.931) |  | 1.225 (1.137-1.317) |  |
| LST (C°): | <50 | 1 | <0.0001 | 1 | 0.4747 | 1 | <0.0001 |
|  | 50-99 | 1.134 (1.074-1.197) |  | 1.027 (0.907-1.161) |  | 1.114 (1.059-1.172) |  |
|  | 100+ | 1.398 (1.295-1.507) |  | 1.122 (0.929-1.343) |  | 1.363 (1.269-1.463) |  |
| Prop forested: | 0-25 | 1 | <0.0001 | 1 | <0.0001 | 1 | <0.0001 |
|  | 25-50 | 1.183 (1.106-1.265) |  | 0.768 (0.656-0.897) |  | 1.109 (1.041-1.180) |  |
|  | 50-75 | 0.844 (0.785-0.907) |  | 0.283 (0.226-0.350) |  | 0.755 (0.705-0.809) |  |
|  | 75-100 | 1.345 (1.262-1.434) |  | 1.453 (1.278-1.653) |  | 1.346 (1.270-1.427) |  |
| Prop cropland: | 0-25 | 1 | <0.0001 | 1 | <0.0001 | 1 | <0.0001 |
|  | 25-50 | 1.208 (1.130-1.292) |  | 0.975 (0.845-1.126) |  | 1.199 (1.127-1.275) |  |
|  | 50-75 | 1.052 (0.982-1.128) |  | 1.136 (0.990-1.304) |  | 1.060 (0.995-1.129) |  |
|  | 75-100 | 1.061 (0.988-1.140) |  | 0.289 (0.229-0.359) |  | 0.948 (0.886-1.015) |  |
| Prop 5km to road: | 0-25 | 1 | <0.0001 | 1 | <0.0001 | 1 | <0.0001 |
|  | 25-50 | 1.166 (1.094-1.242) |  | 1.282 (1.113-1.477) |  | 1.155 (1.089-1.226) |  |
|  | 50-75 | 0.806 (0.753-0.863) |  | 0.812 (0.695-0.947) |  | 0.811 (0.761-0.864) |  |
|  | 75-100 | 0.833 (0.775-0.894) |  | 0.746 (0.630-0.881) |  | 0.833 (0.780-0.890) |  |
| Population density: | 0-25 | 1 | <0.0001 | 1 | <0.0001 | 1 | <0.0001 |
|  | 25-50 | 1.242 (1.159-1.332) |  | 1.309 (1.131-1.515) |  | 1.229 (1.153-1.310) |  |
|  | 50-75 | 1.127 (1.052-1.208) |  | 0.690 (0.583-0.816) |  | 1.048 (0.982-1.118) |  |
|  | 75-100 | 1.189 (1.110-1.274) |  | 1.038 (0.891-1.208) |  | 1.161 (1.089-1.238) |  |
| Elevation (m): | <200 | 1 | <0.0001 | 1 | <0.0001 | 1 | <0.0001 |
|  | 200-499 | 1.572 (1.486-1.663) |  | 1.242 (1.079-1.423) |  | 1.504 (1.425-1.585) |  |
|  | 500+ | 1.591 (1.475-1.714) |  | 2.117 (1.816-2.456) |  | 1.680 (1.569-1.798) |  |
