# Supplementary material for: Designing malaria surveillance strategies for mobile and migrant populations in Nepal: a mixed-methods study
Source: Malar J. 2019 May 3;18:158. doi: 10.1186/s12936-019-2791-1 (PMC6500027; doi:10.1186/s12936-019-2791-1)
Supplement: Supplementary file 2 — Additional file 2. Model selection criteria and model comparison. [file 12936_2019_2791_MOESM2_ESM.docx]

**Designing malaria surveillance strategies for mobile and migrant populations in Nepal: a mixed-methods study**

**Additional file 2. Model selection criteria and model comparison**

All covariates selected via the preliminary analysis described in Additional file 1, the reported cases and catchment population were analyzed along a temporal conditional autoregressive component. Different models were compared using the conditional predictive ordinate (CPO), which is the sum of the predictive log probabilities of the holdout observations given the train set (using leave-one-out cross-validation). Table A2.1 shows a summary of the models compared: Model 1 is a Poisson regression with only fixed effects; Model 2 extends Model 1 by adding an unstructured effects per month and VDC; Model 3 extends Model 1 by adding a seasonal random effect with monthly periodicity and both a structured and an unstructured effects by VDC; and Model 4 is equivalent to model 3 without fixed effects.

The model with highest CPO, and therefore selected as the best model, was Model 3. This model includes a seasonal component to allow the spatial process to evolve according to the observed seasonal month-to-month changes in transmission.

Model 3 is formally defined as follows:

Let $s$ be a spatial index that denotes the VDCs and $t$ be a time index that denotes time measured in months. Indigenous case counts of malaria are assumed to be a Poisson variable such that $y\left( s,t \right) \sim Pois(r\left( s,t \right)E\left( s,t \right))$, where $r$ and $E$ denote the rate and exposure factor respectively. The rate is modelled as a spatio-temporal process of the form

$\log r(s, t)=\alpha+ \sum_{i} \tau_{i}(s,t)+ \sum_{i} \beta_{i}x_{i}\left( s,t \right)+f\left( t \right)+u\left( s,t \right)+v(s,t)$,

where $\alpha$ is the overall mean; $\{\tau_{i}\}$ are factors that include classifications by amount of rain, year, type of district and population density; $\{x_{i}\}$ are varying or invariant covariates with regression parameters $\{\beta_{i}\}$; $f(t)$ is a non linear seasonal effect; and $u(s,t)$ and $v(s,t)$ are the structured and unstructured spatial components of a Besag-York-Mollié (BYM) model with an autoregressive effect (hence the temporal index).

Figure A2. Case counts by month for surveillance data in three districts from 2013-2016, showing the annual periodicity of seasonal transmission. Grey shaded areas indicate times when MMPs report frequent travel back to Nepal.


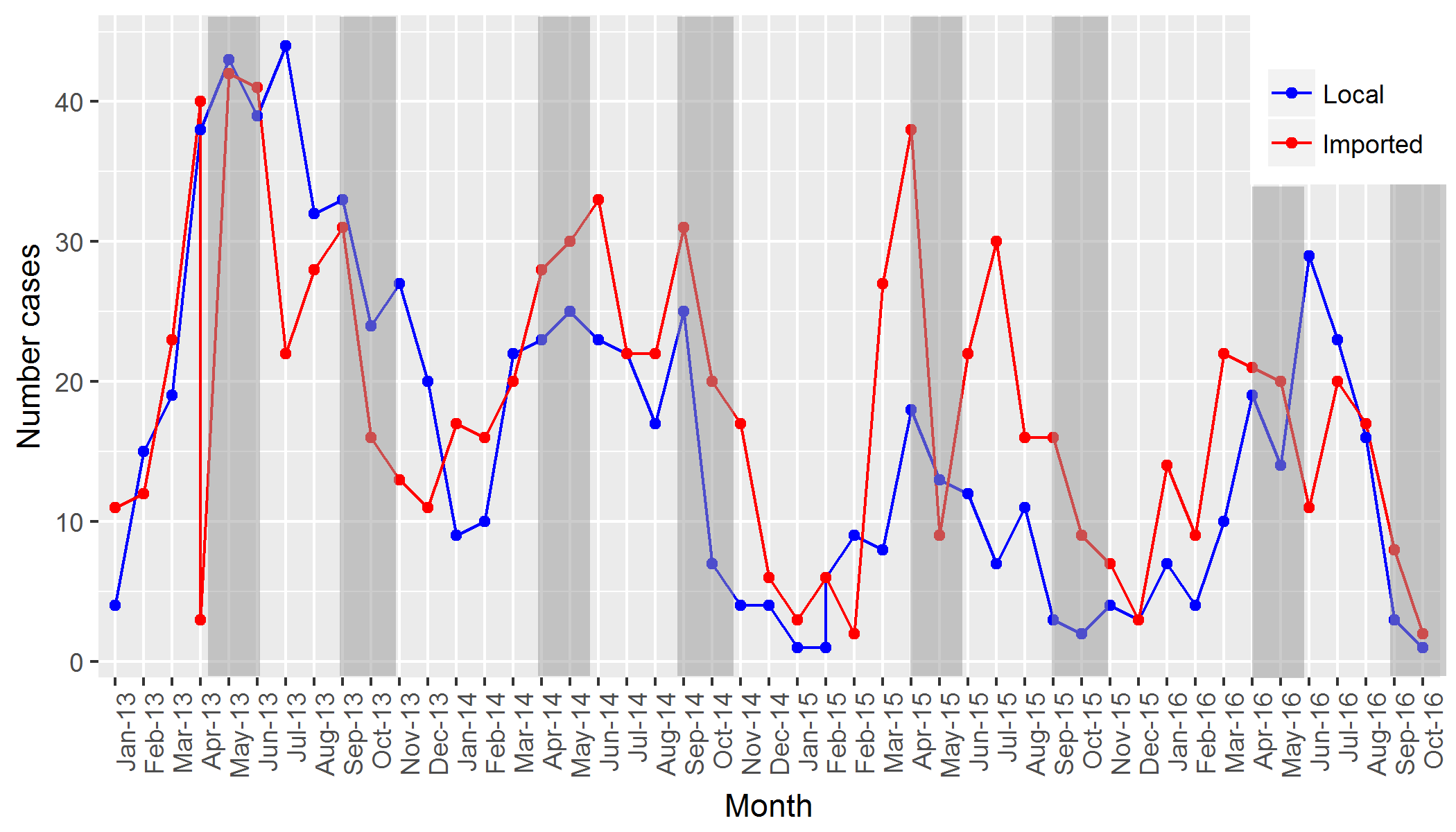


Table A2.1 Comparison of four models of the monthly incidence of indigenous malaria (all species), with odds ratios of fixed effects and precision of random effects.

|  | **Posterior mean, (95% Crl)** | | | | | | |
| --- | --- | --- | --- | --- | --- | --- | --- |
| **Fixed effects (Odds ratios)** | **Model 1**  **(Fixed effects only)** |  | **Model 2**  **(Unstructured RE)** |  | **Model 3**  **(Spatio-temporal, covariates)** |  | **Model 4**  **(Spatio-temporal null)** |
| Lagged importation rate^1^ | 1.038 (1.031, 1.044) |  | 1.015 (1.006, 1.024) |  | 1.020 (1.009, 1.031) |  | - |
| LST (C°) | 1.015 (1.002, 1.029) |  | 1.027 (1.013, 1.041) |  | 1.016 (0.978, 1.049) |  | - |
| Rainfall (mm): <20 | 1 |  | 1 |  | 1 |  | - |
| 20-119 | 1.237 (0.988, 1.556) |  | 1.201 (0.961, 1.507) |  | 1.117 (0.757, 1.618) |  | - |
| 120-1450 | 1.645 (1.333, 2.040) |  | 1.619 (1.315, 2.005) |  | 1.334 (0.8, 2.065) |  | - |
| High population density^2^ | 0.534 (0.449, 0.663) |  | 0.586 (0.371, 0.923) |  | 0.614 (0.399, 0.947) |  |  |
| Year: 2013 | 1 |  | 1 |  | 1 |  | - |
| 2014 | 0.669 (0.555, 0.804) |  | 0.649 (0.538, 0.781) |  | 0.635 (0.48, 0.838) |  | - |
| 2015 | 0.426 (0.335, 0.537) |  | 0.404 (0.317, 0.511) |  | 0.442 (0.305, 0.64) |  | - |
| 2016 | 0.721 (0.581, 0.890) |  | 0.676 (0.542, 0.837) |  | 0.636 (0.436, 0.924) |  | - |
| District: Kailali | 1 |  | 1 |  | 1 |  | - |
| Kanchanpur | 0.596 (0.499, 0.709) |  | 0.494 (0.314, 0.780) |  | 0.475 (0.308, 0.736) |  | - |
| Bardiya | 0.845 (0.649, 1.085) |  | 0.746 (0.466, 1.184) |  | 0.768 (0.486, 1.205) |  | - |
| **Random effects (Precision)** |  |  |  |  |  |  |  |
| Seasonal random effect | - |  | - |  | 2.949 (0.052, 17.118) |  | 0.782 (0.044, 3.234) |
| Month effect unstructured | - |  | 18347.737 (1203.659, 66292.779) |  | - |  | - |
| VDC effect unstructured | - |  | 3.741 (2.01, 6.364) |  | 2.25 (1.464, 3.329) |  | 1.422 (0.988, 1.993) |
| VDC effect structured | - |  | - |  | 3013.549 (745.87, 7326.23) |  | 2983.061 (743.79, 7403.18) |
| DIC | 2033.719 |  | 1860.554 |  | 1810.916 |  | 1816.487 |
| CPO | -1022 |  | -931 |  | -907 |  | -962 |
| Crl Bayesian credible interval; C Celsius; mm millimeter; OR odds ratio; LST land surface temperature; VDC village development committee; DIC deviance information criteria; CPO conditional predictive ordinate  ^1^ Monthly cases per 1000 population, ^2^Defined as the top quartile  Model 1: Fixed effects only; Model 2: Unstructured random effects for month and VDC; Model 3: Spatial VDC random effect within months, allowing for seasonal trend between months; Model 5: Without covariates (Spatial VDC random effect within months, allowing for seasonal trend between months) | | | | | | | |
