# Supplementary material for: Designing malaria surveillance strategies for mobile and migrant populations in Nepal: a mixed-methods study
Source: Malar J. 2019 May 3;18:158. doi: 10.1186/s12936-019-2791-1 (PMC6500027; doi:10.1186/s12936-019-2791-1)
Supplement: Supplementary file 3 — Additional file 3. Comparison of number of cases imported and total number of cases in 2016 reported through case-based surveillance and the World Malaria Report (WMR). [file 12936_2019_2791_MOESM3_ESM.docx]

| Study District | Case-based surveillance | WMR |
| --- | --- | --- |
| Bardiya | 17/32 | 19/33 |
| Kailali | 83/179 | 128/237 |
| Kanchanpur | 47/65 | 51/68 |

**Designing malaria surveillance strategies for mobile and migrant populations in Nepal: a mixed-methods study**

**Additional file 3. Comparison of number of cases imported and total number of cases in 2016 reported through case-based surveillance and the World Malaria Report (WMR)**
