# Supplementary material for: Designing malaria surveillance strategies for mobile and migrant populations in Nepal: a mixed-methods study
Source: Malar J. 2019 May 3;18:158. doi: 10.1186/s12936-019-2791-1 (PMC6500027; doi:10.1186/s12936-019-2791-1)
Supplement: Supplementary file 4 — Additional file 4. Characteristics of focus group participants. [file 12936_2019_2791_MOESM4_ESM.docx]

|  | | | Imported & MMP  N (%) | | Local Cases  N (%) | |
| --- | --- | --- | --- | --- | --- | --- |
| N participants^1^ | | | 43 | (100) | 13 | (100) |
| Participant Type | |  |  |  |  |  |
|  | Imported case |  | 20 | (35.7) | - | - |
|  | MMP contact |  | 23 | (41.1) | - | - |
|  | Local case |  | - | - | 13 | (100) |
| District | |  |  |  |  |  |
|  | Bardiya |  | 0 | (0.0) | 0 | (0.0) |
|  | Kailali |  | 24 | (55.8) | 7 | (53.8) |
|  | Kanchanpur |  | 19 | (44.2) | 6 | (46.2) |
| Gender | |  |  |  |  |  |
|  | Male |  | 39 | (90.7) | 7 | (53.9) |
|  | Female |  | 4 | (9.3) | 6 | (46.2) |
| Age category | |  |  |  |  |  |
|  | 18-24 |  | 20 | (46.5) | 3 | (23.1) |
|  | 25-34 |  | 12 | (27.9) | 7 | (53.9) |
|  | 35-68 |  | 11 | (25.6) | 3 | (23.1) |
| Occupation | |  |  |  |  |  |
|  | Farming |  | 5 | (11.6) | 5 | (38.5) |
|  | Security guard/watchman |  | 7 | (16.3) | 1 | (7.7) |
|  | Factory/Construction |  | 15 | (34.9) | 0 | (0.0) |
|  | Hotel services |  | 2 | (4.7) | 2 | (15.4) |
|  | Housewife |  | 3 | (7.0) | 2 | (15.4) |
|  | Other^2^ |  | 11 | (25.6) | 3 | (23.1) |
| ^1^ Includes data from 9 groups: 5 Kailali and 4 Kanchanpur. Bardiya was excluded due to low case numbers  ^2^ Other category includes students, waiters, army, truck drivers and other types of workers | | | | | | |

**Designing malaria surveillance strategies for mobile and migrant populations in Nepal: a mixed-methods study**

**Additional file 4. Characteristics of focus group participants**
